# Comparative analysis of integrative and conjugative mobile genetic elements in the genus *Mesorhizobium*

**DOI:** 10.1099/mgen.0.000657

**Published:** 2021-10-04

**Authors:** Elena Colombi, Benjamin J. Perry, John T. Sullivan, Amanuel A. Bekuma, Jason J. Terpolilli, Clive W. Ronson, Joshua P. Ramsay

**Affiliations:** ^1^​ Curtin Health Innovation Research Institute, Curtin University, Perth, WA, Australia; ^2^​ Curtin Medical School, Curtin University, Perth, WA, Australia; ^3^​ Department of Microbiology and Immunology, University of Otago, Dunedin, New Zealand; ^4^​ Centre for Rhizobium Studies, Food Futures Institute, Murdoch University, Perth, WA, Australia, Murdoch University, Perth, WA, Australia; ^†^​ Present address: Western Australian Department of Primary Industries and Regional Development, Research and Industry Innovation, South Perth, WA, Australia

**Keywords:** symbiosis, integrative and conjugative elements, ICE, IME, conjugation, mobilization, legumes, bacterial evolution, integrase, recombinase, tripartite ICE

## Abstract

Members of the *Mesorhizobium* genus are soil bacteria that often form nitrogen-fixing symbioses with legumes. Most characterised *Mesorhizobium* spp. genomes are ~8 Mb in size and harbour extensive pangenomes including large integrative and conjugative elements (ICEs) carrying genes required for symbiosis (ICESyms). Here, we document and compare the conjugative mobilome of 41 complete *Mesorhizobium* genomes. We delineated 56 ICEs and 24 integrative and mobilizable elements (IMEs) collectively occupying 16 distinct integration sites, along with 24 plasmids. We also demonstrated horizontal transfer of the largest (853,775 bp) documented ICE, the tripartite ICE*M*spSym^AA22^. The conjugation systems of all identified ICEs and several plasmids were related to those of the paradigm ICESym ICE*Ml*Sym^R7A^, with each carrying conserved genes for conjugative pilus formation (*trb*), excision (*rdfS*), DNA transfer (*rlxS*) and regulation (*fseA*). ICESyms have likely evolved from a common ancestor, despite occupying a variety of distinct integration sites and specifying symbiosis with diverse legumes. We found extensive evidence for recombination between ICEs and particularly ICESyms, which all uniquely lack the conjugation entry-exclusion factor gene *trbK*. Frequent duplication, replacement and pseudogenization of genes for quorum-sensing-mediated activation and antiactivation of ICE transfer suggests ICE transfer regulation is constantly evolving. Pangenome-wide association analysis of the ICE identified genes potentially involved in symbiosis, rhizosphere colonisation and/or adaptation to distinct legume hosts. In summary, the *Mesorhizobium* genus has accumulated a large and dynamic pangenome that evolves through ongoing horizontal gene transfer of large conjugative elements related to ICE*Ml*Sym^R7A^.

## Data Summary

Bacterial genomes analysed in this study have been deposited previously in the National Centre for Biotechnology Information (NCBI) Genome database and are listed in Table S1 (available with the online version of this article). Genomes sequences produced in this study are deposited in NCBI, accession number NZ_NSFP00000000 and CP048406. Nine supplementary figures and seven supplementary tables are included in the online version of this article. Details on the programmes and scripts used are available at https://github.com/EC-Rufina/MesICE.

**Impact Statement**

Nitrogen-fixing symbioses between legumes and rhizobia are a major contributor to global nitrogen cycling. For rhizobia of the genus *Mesorhizobium *, genes required for symbiosis are usually encoded on horizontally transmissible integrative and conjugative elements (ICEs). ICEs are important components of the bacterial accessory genome and key drivers of bacterial evolution but remain relatively unexplored compared with their plasmid counterparts. In this study, we performed a comparative analysis of mobile genetic elements identified in 41 complete *Mesorhizobium* genomes to gain insight into ICE distribution, evolution and structure. ICEs carrying symbiosis genes (ICESyms) likely evolved from a common ancestor and have subsequently diverged to integrate at a variety of chromosomal sites and specify symbiosis with several distinct legume hosts. Overall, the *Mesorhizobium* mobilome was dominated by ICEs and conjugative plasmids harbouring conjugation-gene clusters related to those of ICESyms and we observed extensive evidence of recombination between elements. Frequent rearrangement and duplication of genes controlling quorum sensing and ICESym transfer regulation suggested ICEs are undergoing extensive evolution to fine-tune their transferability. This study provides a significant advance in our understanding of *Mesorhizobium* genome organization and ICESym evolution.

## Introduction

Horizontal gene transfer (HGT) is a major force shaping bacterial evolution [1–5]. Transfer of suites of genes encoding metabolic pathways refined by selection in a donor organism can endow recipients of HGT with the ability to colonise new ecological niches in a single evolutionary step [6–8]. Genome analyses suggest the most abundant type of conjugative elements in prokaryotes are integrative and conjugative elements (ICEs) [9]. ICEs are chromosomally-integrated elements that are passively replicated as a part of the genome but are capable of horizontal transmission facilitated by their encoded excision and conjugation systems [10, 11]. ICEs typically carry a conserved set of ‘backbone’ genes essential for ICE integration, excision, conjugation and regulation of transfer [12]. In addition to backbone genes, ICEs often carry accessory genes of adaptive significance to their hosts, such as genes affecting biofilm formation, pathogenicity, antibiotic and heavy metal resistance, bacteriocin synthesis, iron acquisition, and symbiosis [13–23]. During conjugative transfer, ICEs excise from the chromosome and circularise. ICE DNA is then nicked by a conjugative relaxase, and a single-stranded DNA copy of the ICE is transferred to the new host. Second strand synthesis, recircularization, and integration then occurs in donor and recipient cells [10, 11]. The conjugative machinery of ICEs and conjugative plasmids can also facilitate horizontal transfer of co-resident non-conjugative elements such as mobilizable plasmids and integrative mobilizable elements (IMEs) [24].

Gram-negative soil bacteria in the genus *Mesorhizobium* can establish a non-obligatory nitrogen-fixing symbiosis with legumes [25]. Symbiotic *Mesorhizobium* spp. infect legume root nodules, within which the bacteria fix atmospheric N_2_ into NH_3_, which is assimilated by the plant. In return the host plant supplies carbon, essential nutrients, and a niche for the microsymbiont. In *Mesorhizobium* spp.*,* genes essential to the establishment of N_2_-fixing associations are most often encoded on symbiosis ICEs (ICESyms) [19, 26–28] and occasionally on plasmids [29]. Symbiosis ICEs capable of horizontal transfer have been described in *Mesorhizobium* spp. isolated from nodules of *Lotus*, *Biserrula pelecinus* (biserrula) and *Cicer* (chickpea) [19, 26–28, 30]. ICESyms have been observed to transfer to non-symbiotic mesorhizobia both in natural settings and laboratory conditions, converting them into symbionts [19, 26, 30].

ICE*Ml*Sym^R7A^ was the first ICE described in *Mesorhizobium* spp. and it confers upon its host the ability to form N_2_-fixing symbiosis with *Lotus* spp. [31]. The regulatory network controlling excision, conjugation and regulation of the ICE has been described in detail [32–36]. ICE*Ml*Sym^R7A^ excision and integration is catalysed by the site-specific recombinase IntS. During integration of ICE*Ml*Sym^R7A^ into the recipient chromosome, IntS catalyses recombination between a DNA site called *attP* on the circularised ICE and a corresponding *attB* site on the host chromosome, which for ICE*Ml*Sym^R7A^ is the 3′ end of the tRNA-Phe gene [19]. Integrase-mediated recombination between short identical ‘core’ sequences within *attP* and *attB* results in the integration of the ICE into the chromosome and the creation of two new attachment sites *attL* and *attR*, which flank the integrated ICE. Recently, ICESyms with a tripartite structure were described in *Mesorhizobium* spp. [26]. The tripartite ICE ICE*Mc*Sym^1271^ confers an ability to form a symbiosis with *Biserrula pelecinus*. ICE*Mc*Sym^1271^ and other tripartite ICEs possess three distinct site-specific integrases *intS*, *intM* and *intG,* that sequentially recombine three separate chromosomal regions α, β and γ, through three pairs of *attL* and *attR* sites, to form a single circular DNA element for conjugative transfer [37]. The tripartite α regions are the largest fragments of tripartite ICEs, and while the α regions lack integrase genes (which are encoded on fragments β and γ) they appear to carry most genes with known involvement in symbiosis and conjugative transfer.

The aim of this work was to characterise the mobilome of *Mesorhizobium* spp. in order to gain insight into its evolution. The accurate delineation of ICEs and large tripartite ICEs that frequently exceed 0.5 Mb in length requires complete and accurately assembled genomes. In this work, we interrogated 41 complete *Mesorhizobium* genome sequences and conducted a comparative analysis of conjugative and mobilizable elements. The *Mesorhizobium* spp. mobilome was found to be dominated by ICEs and conjugative plasmids with conjugation and regulatory genes related to ICE*Ml*Sym^R7A^. The various ICESyms specifying symbiosis with diverse legumes [26–28, 30] have likely evolved from a common ancestor within the larger family of proteobacterial ICEs [34, 38]. Extensive evidence for gene exchange between mobile elements suggests that ICEs, IME and plasmids have frequently recombined, swapped genetic cargo and their mechanisms of host maintenance. Evidence of frequent rearrangement and duplications of quorum-sensing regulatory genes controlling the induction of ICESym mobilization suggest that the rates of ICE transfer are under continuing selection.

## Methods

### Whole genome sequencing

*Mesorhizobium* sp. AA22 was sequenced both using Illumina HiSeq 2×100 bp paired-end reads (MrDNA) and the PacBio RSII platform (Macrogen, South Korea). PacBio raw reads were *de novo* assembled with Flye 2.6 [39]. The assembly was polished with the Pacbio reads five times using Minimap2 2.17-r941 [40] and Racon 1.4.3 [41]. The assembly was then further polished five times with the Illumina HiSeq reads using Pilon 1.23 [42]. The starting position of the genome was set with Circlator 1.5.5 [43].

The transconjugant strain *M. japonicum* R7ANSxAA22 was sequenced using Illumina MiSeq. Illumina sequence adapter contamination was removed using nesoniclip (v0.132) (https://github.com/Victorian-Bioinformatics-Consortium/nesoni), and reads were corrected using Lighter (v1.1.1) [44] and assembled with SPAdes V. 3.5.0 [45].

### *Mesorhizobium* genus phylogeny

The *Mesorhizobium* genus phylogeny was constructed using a method modified from Prokchorchik *et al*. [46]. Genomes (Table S1) were re-annotated using Prokka [47], and Proteinortho [48] was run with default settings to identify single-copy core genes. The nucleotide sequences of 1,609 single copy orthologues were then aligned with the MAFFT algorithm [49], and core gene alignments were concatenated and stripped of gaps with Goalign (https://github.com/evolbioinfo/goalign). Maximum likelihood phylogeny reconstruction was performed with RAxML v8.2.10 (parameters: -f a -p $RANDOM -x $RANDOM -N 100 m GTRCATX -T 16) [50]. The tree was rooted at midpoint using the midpoint() function from the phangorn package in R [51] and then it was visualised using FigTree v1.4.4 (https://github.com/rambaut/figtree/), only nodes with bootstrap values above 80 were displayed (Fig. S1).

Pairwise average nucleotide identity (ANI) was calculated using FastANI [52] on the entire genome of the strains. Strains were classified as same species if they showed an ANI of 95 % or above [53].

### Mobile genetic element search and pangenome classification

To identify putative horizontally transmissible elements, relaxase gene sequences were searched with hmmscan from HMMER [54] using available hidden Markov model (HMM) protein profiles (MOB database) [55] (Table S2) defined for distinct MOB-protein families [56]. For this search, a bit score threshold (-T) of 33 was used. For ambiguous classifications, the MOB-protein family was chosen by the best e-value in the full sequence. For the identification of ICEs and IMEs, genomes were queried using the blastn algorithm [57] using *trbB* (EB234_29245) from ICE*Ml*Sym^R7A^ [58], and *traA* (EB235_34495) from ICE*Ml*adh^R88B^ [59], here referred as IME*Ml* ^R88b^. We used a cut off of 50 % pairwise identity and, where applicable, only elements with recognisable *att* sites were selected (Table S3). Four pTONO-1-like sequences were present within chromosomal contig assemblies (CP034446.1, CP034447.1, CP034448.1, and CP034451.1). While it was possible that these plasmids were indeed chromosomally integrated, this seemed unlikely as each of the regions harboured plasmid-like replication genes (*repABC*), and *att* sites were not detected, suggesting the plasmid sequences were artificially joined to the chromosome sequence during assembly (Table S2).

The tool Phaster [60] was used to search all the genomes for potential prophage regions. Regions classified as 'intact' were inspected manually for the presence of capsid and tail proteins. Phage boundaries were delineated by the identification of direct sequence repeats (part of *att* core regions) produced following the integration of the phage in the genome (Table S3).

Core and accessory genome calculations were initially performed using iterative incrementation of the minimum percent identity threshold of BLASTP for the assignment in protein ortho-groups from 20–95 % amino-acid identity (Fig. S2); to avoid underestimating the number of accessory gene orthologues, Proteinortho was run with default settings (25 % identity) and reporting singleton genes.

### Backbone identification

Backbone genes were identified as genes present in at least 95 % of the elements, a threshold that was introduced to take into account sporadic deletion events. The identification of backbone genes was guided by Roary [61] and Proteinortho [48]. For Roary, a minimum amino-acid identity of 40 % was used because with this cut-off the programme identified genes known to be involved in the ICE life cycle. Proteinortho was run with default settings. Both programmes identified the same set of conserved genes.

### ICE comparative analysis

For all the following analyses, only non-redundant ICEs (Table S3) were used. Redundancy was defined based on overall gene content rather than by accumulation of SNPs, i.e. an element was considered redundant if it shared the same accessory genes as another ICE, with the exception of insertion sequences (IS). The nucleotide sequences of strictly conserved and single-copy backbone genes (Table S4) were aligned with the MAFFT algorithm [49], trimmed and concatenated in Geneious [62]. The alignment was used to build phylogeny of the elements using PhyML 3.3 [63] with 100 bootstrap replicates which was rooted at midpoint using the midpoint() function from the phangorn package in R [51]. This alignment was also used to build a neighbor-net tree with SplitTree [64], and to infer recombination with ClonalFrameML [65]. For the integrase and quorum sensing genes trees, nucleotide sequences were aligned with the MAFFT algorithm [49], the tree built with RAxML v8.2.10 (parameters as above) [50], and visualised with FigTree (v.1.4.4, http://tree.bio.ed.ac.uk/software/figtree/).

The *nod* box site motifs were identified as described in Perry *et al*. [28]. nhmmscan [66] was run using the training sets of previously characterised *nod* box sequences from ICE*Ml*Sym^R7A^ and ICE*Ml*Sym^NZP2037^ [58, 67] as models.

Alfy [68] was used to identify chimaerism among ICEs and IMEs. Alfy was run selecting only matches with a *P* value <0.05 within a sliding window of 1000 bp. Cytoscape [69] was then used to visualise the percentage of recombined sequences among ICEs, IMEs, and plasmids.

A distance matrix was calculated with the dist.gene() function from the ape package in R [70] using as input the gene presence-absence data produced by Roary run with a minimum amino-acid identity of 40 %. The distance matrix was then used to build a neighbor-joining tree using the nj() function from the ape package in R [70]. eggNOG mapper 5.0 [71] was used for the functional annotation of accessory genes. Pangenome-wide association analyses of the ICEs were performed with Scoary [72]. *Mesorhizobium* sp. M1D.F.Ca.ET.043.01.1.1 harbours two ICESyms that carry redundant symbiosis genes and one of the two ICESyms (ICEMsp.Sym^M1D^-1) appeared to be undergoing pseudogenization; for example it carried a truncated copy of *nodC* [27]. ICEMsp.Sym^M1D^-1 was therefore removed to avoid misleading the association analysis. To identify genes associated with symbiosis, Scoary was run on the output files produced by Roary that was run on all elements with minimum percentage identity for BLASTP of 40 % and with paralogs not split. For the identification of genes associated with the host plant, Roary was run on only ICESyms using a minimum percentage identity for BLASTP of 70 % and with paralogs not split. Full list of genes associated with symbiosis ICEs and associated with the host of isolation are reported in Tables S5 and S6 respectively.

Searches for antimicrobial resistance genes were performed using ResFinder v3.1 (https://cge.cbs.dtu.dk/services/ResFinder/), searches for bacteriocins and non-ribosomally synthesized and post-translationally modified peptides were performed with Bagel4 [73], and CRISPR-Cas systems with CRISPRDetect [74].

### Conjugation experiments

*M. japonicum* R7ANS pFAJ1708-GFP [26] was used as recipient strain and *Mesorhizobium* sp. AA22 as donor strain. R7ANS is auxotrophic for biotin, nicotinate and thiamine, and the genes for the biosynthesis of biotin and nicotinate are present on ICE*M*sp.Sym^AA22^, so exconjugants were selected on defined (G/RDM) medium containing thiamine (1 µg ml^−1^) and tetracycline (2 µg ml^−1^), but lacking biotin and nicotinate. Donor and the recipient were separately grown to stationary phase in liquid TY at 28 °C with shaking at 200 r.p.m. One millilitre of culture of donor and recipient strains were pelleted by centrifugation at 14,000 r.p.m. for 1 min, resuspended together in 50 µl of TY, spotted onto TY agar plates, and incubated at 28 °C for 48 h. Spot matings were resuspended in sterile deionized water and serial dilutions were plated onto G/RDM supplemented with tetracycline and thiamine to select transconjugant strains, and onto G/RDM supplemented with thiamine to count donor strains. Plates were incubated at 28 °C for 7–10 days. The rate of conjugative transfer was calculated as the number of transconjugants per donor cell.

## Results and discussion

### Characterisation of complete *Mesorhizobium* genomes

Forty complete or nearly complete *Mesorhizobium* spp. genomes (Table S1) available from NCBI (updated April 2020) were analysed. The complete genome sequence of the *B. pelecinus* ssp. *pelecinus* symbiont *Mesorhizobium* sp. AA22 [75] was additionally completed in this study. A maximum-likelihood phylogeny inferred from a concatenated alignment of single-copy core genes revealed the 41 strains represented a genetically diverse group of mesorhizobia, and their ANI score showed they comprise 29 genospecies (Fig. S1). Most genomes analysed here are from strains isolated from root nodules of legume hosts *Cicer*, *Lotus* or *Biserrula* but also included are single strains isolated from *Astragalus sinicus* and *Robinia pseudoacacia*. Seven strains are from soil or aquatic environments.

Previous large-scale genome comparisons have revealed mesorhizobia harbour large and plastic pangenomes [27, 28]. Iterative pangenome analyses were carried out here with the 41 complete genomes using Proteinortho [48], initially using protein identity cut-offs ranging from 20–95 % (Fig. S2). The estimated number of core genes remained largely unchanged with cut-off values between ~20–60 %, but decreased above 60 %, suggesting values <60 % include most core genes without counting core genes from more distantly-related species as distinct orthologues. Using a 25 % cut-off, the average genome in this dataset encoded 6386 proteins of which 1670 (26 %) were part of the core genome.

### Identification of ICEs, IMEs, conjugative plasmids and mobilizable plasmids in complete *Mesorhizobium* genome sequences

**Fig. 1.**
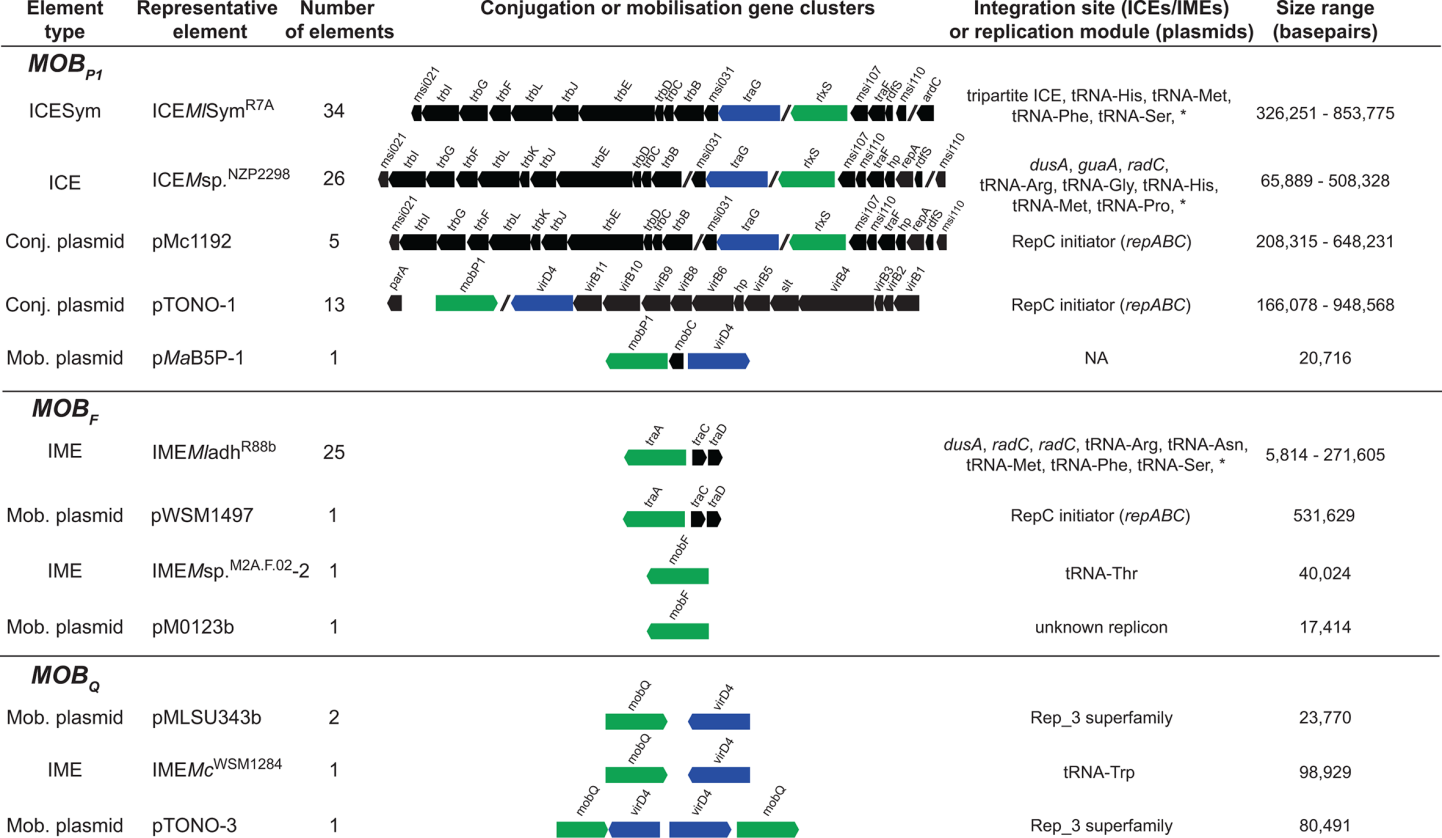
Representative gene maps of identified conjugation and mobilization regions. For each family of MOB relaxase, a representative element of each type (ICE, IME, conjugative or mobilizable plasmid) is shown and the number of elements of the same type found in the 41 genomes is indicated. The gene map of the conjugative genes and relaxase locus displayed refers to the representative element: in black conserved genes predicted to have a role in conjugation, blue and green boxes depict T4CP and relaxase genes, respectively. Relaxases were named after their family, unless previously named. Replication modules carried by plasmids and integration sites occupied by ICEs or IMEs are reported, with * indicating *att* sites were not identified for some integrative elements.

Conjugative DNA relaxases - also referred to as ‘Mob’ proteins - are DNA endonucleases that target and nick the origin-of-transfer sequences present on mobile genetic elements. Relaxases are required for recruitment of ssDNA of conjugative and mobilizable elements to the conjugative type IV secretion system (T4SS) for transfer. Relaxase proteins have been assigned into six MOB families: MOB_F_, MOB_H_, MOB_Q_, MOB_C_, MOB_P_ and MOB_V_ [76]. MOB_P_ is the largest family and includes the TraI relaxase of the broad-host range plasmid RP4; the MOB_F_ family includes the TraI relaxase of the F plasmid, and MOB_Q_, which partially overlaps with the MOB_P_ family, includes various tumour-inducing (Ti) plasmids of *Agrobacterium* (not to be confused with VirD2 relaxases) and relaxases encoded by several rhizobial plasmids. Potential conjugation and conjugative mobilization systems in the *Mesorhizobium* genomes were identified by searching for each of the MOB-family proteins using hidden-Markov models [55]. Eighty-two MOBP subtype 1 (MOB_P1_) relaxase genes were identified on plasmids and chromosomes, including the ICE*Ml*Sym^R7A^ relaxase RlxS (Table S2, Fig. 1). blastn analysis performed using the ICE*Ml*Sym^R7A^ type IV secretion system (T4SS) conjugation gene *trbB* (EB234_29245) revealed 60 chromosomal regions and five plasmids carrying ICE*Ml*Sym^R7A^-like T4SS conjugation genes, which resemble the *trb* conjugation-gene clusters of plasmid RP4 [58, 77, 78]. Thirteen MOB_P1_ genes were located on plasmids with distinct *vir-*like conjugative T4SS gene clusters resembling those of the *Mesorhizobium* sp. TONO plasmid pTONO-1. One of the MOB_P1_ plasmids (pMaB5P-1) carried a T4SS coupling-protein (T4CP) gene *virD4* and two *mobP_1_C* loci, but no T4SS genes, suggesting it was potentially mobilizable by other conjugative elements.

Twenty-nine MOB_F_ relaxase genes were identified in chromosomal sequences and two on plasmids. The majority of the MOB_F_ genes (25 IMEs and one plasmid across 17 genomes) were part of a Ti-plasmid-like *traACD* locus containing genes for the TraA relaxase and the relaxasome accessory factors TraC and TraD [79]. The *traA* and *traC* genes on each element were divergently orientated, as they are on the Ti plasmid where they flank the origin of transfer sequence (*oriT*). This *traACD* locus was previously identified on an adhesin-encoding IME located downstream of ICE*Ml*Sym^R7A^ in *Mesorhizobium* sp. R88B [59, 80] (renamed here IME*Ml* ^R88B^, consistent with its classification as an IME). Each of the IME*Ml* ^R88B^-like *traACD* regions additionally carried a putative recombination directionality factor (RDF) gene downstream of *traA*, suggesting that RDF-gene transcription and chromosomal excision of the IME may be transcriptionally coupled with relaxase-gene expression. The remaining four chromosomal MOB_F_ relaxases were associated with unrecognised elements. One IME (IME*M*sp.^M2A.F.02^-2) carrying a lone MOB_F_ gene was also identified. Only five MOB_Q_ relaxase-genes identified, four were located across three plasmids and one on a putative IME (Table S2, Fig. 1). All putative mobilizable plasmids and IMEs that lacked *traACD* genes carried one or more copies of a *virD4*-like T4CP gene. No IME carried complete set of nod-factor and nitrogenase-biosynthesis genes required for nitrogen-fixing symbiosis.

To delineate the chromosomal boundaries of identified ICEs and IMEs, sequence regions surrounding the MOB genes and/or conjugation-gene clusters were inspected for integrase genes and flanking DNA sequence repeats commonly present within integrase attachment (*att*) sites. For tripartite ICEs, the conserved *att* site core sequence repeats corresponding with the integrases IntS, IntG and IntM were used both to identify the presence of tripartite ICEs and delineate the three tripartite ICE regions in the chromosome. In total, 56 ICEs across 38 genomes were delineated (Table S3). The *att* sites were not identified for four ICEs (Table S2). Delineated ICEs ranged in length from 65,889 bp for ICE*Ml* ^NZP2042^, to 853,775 bp for the tripartite ICE, ICE*M*spSym^AA22^. All ICEs identified carried ICE*Ml*Sym^R7A^-related conjugation-gene clusters. Genes for nitrogen fixation and symbiosis were identified on 34 ICEs and these are hereafter collectively referred to as ICESyms. Thirteen of the ICESyms were tripartite in structure and carried the same three pairs of *att* sites and integrase genes as the paradigm tripartite ICE ICE*Mc*Sym^1271^. Monopartite ICESyms of *Mesorhizobium* spp. isolated from *Lotus* were all integrated adjacent to the tRNA-Phe gene, while monopartite *Cicer* ICESyms were integrated adjacent to a tRNA-Ser gene (Table S3) as documented for *M. ciceri* CC1192 [30]. ICEs lacking symbiosis genes were identified adjacent to tRNA-Met, tRNA-Arg, tRNA-Gly, tRNA-His, and tRNA-Pro genes, as well as *guaA*, *radC* and *dusA*. Chromosomal boundaries for 24 putative MOB_F_-carrying IMEs (Table S3) were delineated. IMEs ranged in size from 5,814 bp (IME*M*sp.^M2A.F.05^-1) to 271, 605 bp (IME*M*sp.^NZP2077^-1). Three IMEs were integrated adjacent to a tRNA-Asn gene, while the remaining 21 utilised the same spectrum of *att* sites as ICEs (*radC*, tRNA-Met, tRNA-Arg, tRNA-Phe and tRNA-Ser genes). IME*M*sp.^M2A.F.02^-2 (40, 024 bp) and the MOB_Q_-carrying IME, IME*Mc* ^WSM1284^ (98, 929 bp), occupied unique sites adjacent to tRNA-Thr and tRNA-Trp genes, respectively. In total, the 79 identified ICEs and IMEs collectively occupied 16 distinct integration sites within the 41 *Mesorhizobium* genomes (Table S7). Seventy-three of the elements carried integrase genes encoding tyrosine recombinases and serine recombinases/integrases were present on five IMEs and one ICE.

Five of the 13 conjugative plasmids identified carried conjugation-gene clusters related to that of ICE*Ml*Sym^R7A^ and the remaining eight carried conjugation-gene clusters resembling the *vir*-like T4SS system of pTONO-1 (Fig. 1). Conjugative plasmids with ICESym-like T4SS genes ranged in size from 208,315 bp (pMLb of MAFF303099) to 648,231 bp (pMc1192 of CC1192) and none of these carried a complete set of nodulation or nitrogen-fixation genes. pMc1192 was recently shown to be not essential for N_2_ fixation with chickpea [30]. Overall, most genomes contained at least one ICE (1.22±0.85 ICE per genome) and less frequently IMEs (0.5±0.7 IME per genome). Twenty-five genomes contained no plasmids, nine contained a single plasmid and seven harboured multiple plasmids. *M. oceanicum* sp. B7, *M*. sp. 8, *M. amorphae* CCNWGS0123 and *M*. sp. 7653R were the only strains lacking any detectable ICEs or IMEs and, interestingly, CCNWGS0123 and 7653R were also the only strains carrying a complete complement of genes required for nodulation and nitrogen fixation on plasmids. Only the symbiosis plasmid of CCNWGS0123 carried a conjugation-gene cluster (*vir-*like).

The identified mobile genetic elements (MGE) carry approximately 12 % the identified pangenome. This suggests a major fraction of the pangenome is either located on unidentified MGE or that most of the pangenome genes have lost mobility and now form a large but stable fraction of each chromosome. For the pangenome genes identified on MGEs, i.e. the mobile pangenome or mobilome, ~72 % were present on ICEs and of these, 78 % were present on ICESyms (Fig. S3). Plasmids and IMEs carried 20.4 and 5.9 % of the mobilome, respectively, while bacteriophages carried only 4.6 %.

### ICEs in *Mesorhizobium* share a conserved core-gene organisation

**Fig. 2.**
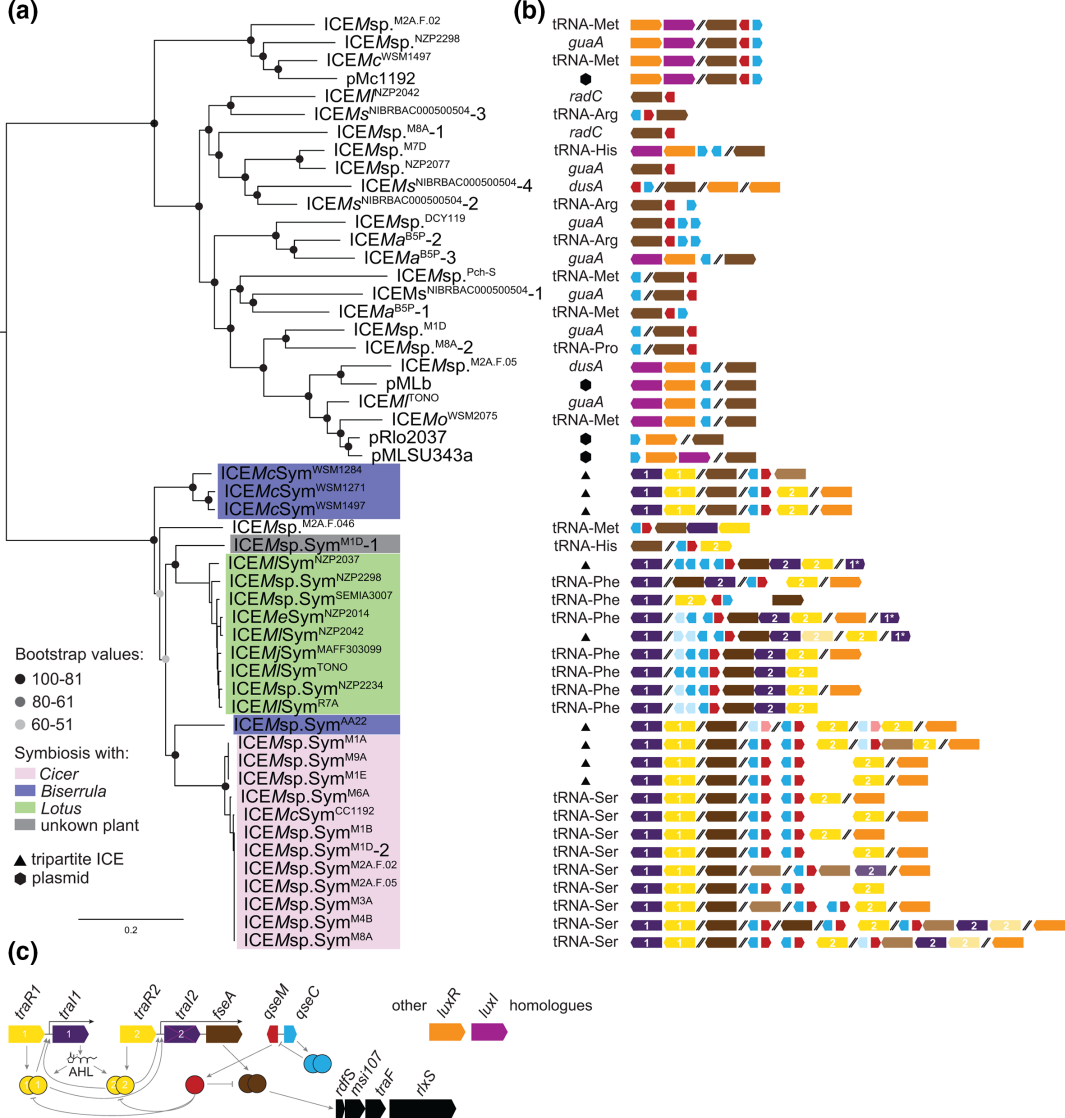
ICE phylogeny and regulatory-gene organisation. (**a**) PhyML tree based on the concatenation of alignments of 15 single-copy backbone genes, scale bar indicates substitutions per site. (**b**) Organization of regulatory genes colour-coded as in *c*, lighter boxes are likely pseudogenes, numbers in *traR* and *traI* indicate the relatedness between the copies as described in main text, * indicates the gene is truncated. (**c**) Regulatory network activating ICE transfer in ICE*Ml*Sym^R7A^ and ICE*Mc*Sym^1271^.

Proteinortho [48] and Roary [61] were used to identify protein-coding genes conserved across 95 % of ICEs and plasmids carrying an ICE*Ml*Sym^R7A^-like conjugation-gene cluster. Both tools identified 17 backbone genes including the ICE*Ml*Sym^R7A^ conjugation-gene cluster (T4SS) genes *msi031, trbBCDEJLFGI* and *msi021*, the T4CP gene *traG*, the relaxase *rlxS*, the putative peptidoglycan transglycosylase gene *msi107*, the *traF* prepilin peptidase gene and the RDF gene *rdfS*. Genes encoding the DUF736 domain (*msi110* on ICE*Ml*Sym^R7A^) were present on all elements (Fig. S4), often in duplicate. Concatenated DNA alignments of the single-copy backbone genes (Table S4) were used to generate a maximum likelihood tree (Fig. 2a). Despite the association of ICESyms with a variety of integrase genes and chromosomal integration sites, the tree grouped all monopartite and tripartite ICESyms into a single clade that exhibited lower average sequence divergence (88 % pairwise nucleotide identity) than other ICEs (69 % pairwise nucleotide identity). Only one ICE lacking symbiosis genes (ICE*M*sp.^M2A.F.046^) grouped with the ICESyms. ICESyms present in strains isolated from the same plant genus grouped together (with the exception of ICE*M*sp.Sym^AA22^), even though alignments used to produce the tree only included genes with transfer-related functions. Interestingly, all identified ICESyms (and ICE*M*sp.^M2A.F.046^) lack the conjugation entry exclusion gene *trbK* [58, 81], genes encoding a putative Rep_3 superfamily replication initiator, and DUF2840-domain protein located between *rdfS* and *traF* (Figs 1 and S4), which were present on all other ICEs identified. These genes are present on distantly related ICEs and plasmids found throughout the proteobacteria, including Tn*4371* [34, 38], suggesting gene loss occurred in a common ancestor of the ICESyms. Conjugative plasmids pMLb and pCC1192 clustered closely with ICEs present in separate branches of the tree, suggesting the conjugation-gene clusters on these elements may have switched between ICE and plasmid modes of host maintenance. All plasmids carrying an ICE*Ml*Sym^R7A^-like conjugation system retained the *rdfS* gene, which in ICE*Ml*Sym^R7A^ and ICE*Mc*Sym^1271^ stimulates integrase-mediated ICE excision [32, 37]. RdfS is also a transcriptional regulator [36, 37], so it may have additional regulatory roles in transfer, which may explain its retention on plasmids [9].

Comparisons of ICE backbone gene phylogeny and ICE integration site suggested there was no strict association between the two. The phylogenies of tripartite ICE integrase genes (Fig. S5) and their ICE backbone genes (Fig. 2a) were also incongruent. While the tripartite ICEs carry closely related IntG and IntM integrases that group separately from similar integrase genes on monopartite ICEs, the transfer genes of monopartite and tripartite ICEs present in *Lotus*-associated mesorhizobia are more closely related to each other than they are to those of tripartite ICEs present in *Cicer*-associated mesorhizobia and vice versa.

### Gene duplications and pseudogenization have shaped quorum sensing and transfer regulation on ICESyms

Excision and conjugative transfer of ICE*Ml*Sym^R7A^ and the tripartite ICE, ICE*Mc*Sym^1271^, are activated by quorum sensing; however, they carry different arrangements of *traR* and *traI* genes. ICE*Ml*Sym^R7A^ carries a single copy of the QS regulator gene *traR* (or *msi174*, referred to here as *traR2*) and two acyl-homoserine lactone synthase genes named *traI1* and *traI2*. While the TraI2 sequence shares 65 % amino-acid identity with TraI1, the *traI2* gene is not essential for AHL production and an in-frame deletion of *traI2* is not affected for ICE*Ml*Sym^R7A^ excision or transfer [33]. Conversely, ICE*Mc*Sym^1271^ lacks *traI2* altogether, but carries two functional copies of *traR* (*traR1* and *traR2*) encoding proteins with 54 % amino-acid identity, and a single copy of *traI* (*traI1*). Here we searched for TraR/TraI homologues encoded on ICESyms, which revealed further variations in copy number and gene organisation of the *traR* and *traI* genes (Figs 2b and S6). While additional genes encoding TraR/LuxR-family regulators were also identified both on ICESyms and other ICEs and plasmids, the predicted proteins were distantly related (~25 % amino-acid identity). All but one ICESyms and ICE*M*sp.^M2A.F.046^ carried *traI1* but mostly only *Lotus*-associated ICESyms carried a copy of *traI2*. No *Lotus*-associated ICESyms carried a copy of *traR1*, but closer inspection revealed that a ~100 bp remnant of the 3′ end of *traR1* was present directly upstream of each *traI1*, confirming that an ancestral copy of *traR1* had been deleted in this ICE lineage. Nucleotide alignments of the eight *traI2* and eight *traI1* genes on *Lotus*-ICESyms revealed that *traI2* sequences collectively carried 147 unique SNPs, 92 amino-acid changes and two frame-shifting deletions. In contrast, *traI1* genes exhibited lower divergence with only 23 unique SNPs (13 synonymous). This suggests the *traI2* alleles, while conserved on *Lotus*-associated ICESyms, are at various stages of pseudogenization. These observations support our previous proposal [37] that gene duplication of the *traR-traI* locus occurred in an ancestor of the ICESym clade and subsequently different ICESyms have variably lost one of the duplicates.

On ICE*Ml*Sym^R7A^, both TraR2 and the activator of *rdfS* expression, FseA, are inhibited by the antiactivator QseM [35]. *qseM* expression is controlled by an adjacently-encoded helix-turn-helix protein QseC (Fig. 2c). Searches for *qseM* and *qseC* genes on ICEs revealed numerous duplications and rearrangements. ICESyms of *Lotus* and *Biserrula* each harboured one copy of *qseM* and multiple variants of *qseC*. In some instances, *qseC* variants carried mutations and/or deletions likely rendering them non-functional (Fig. 2b). All *Cicer* lineage ICEs harboured at least two distinct copies of paired *qseM-qseC* genes, with the exception of ICE*M*sp.Sym^M2A.F.02^. The presence of two copies of *qseM* in the *Cicer*-associated ICESyms suggests that there may be distinct inputs controlling each *qseM* gene, or perhaps the two QseM proteins may target the two TraR proteins also encoded by these ICEs with differing affinities. In summary, both positive and negative regulators controlling QS and conjugative transfer have been frequently duplicated, pseudogenized and rearranged on ICESyms, suggesting that natural selection is constantly adjusting the regulation of ICESym transfer.

### Cargo genes of conjugative and mobilizable elements

Functional annotation by eggNOG mapper [71] of the accessory genes on ICESyms indicated 56 % had putative metabolic roles. Of these, 27 % were likely involved in amino-acid metabolism and transport, 24 % in energy production, and 16 % in coenzyme metabolism. Similarly, a major portion of the accessory genes (43 %) carried by other ICEs and related plasmids were predicted to be involved in metabolism, including 28 % potentially involved in to amino-acid metabolism and transport, and 25 % in energy production and conversion. Collectively ICESyms carried 11,726 genes, with 2,200 (19 %) genes present on one ICE only. Only 66 accessory genes were present on all ICESyms, while no accessory genes were universally conserved ICEs lacking symbiosis genes, which collectively encoded 4,037 genes, of which 1,830 were present on one ICE only (45 %). A similar pattern was observed for IMEs, which collectively carried 3,195 accessory genes, 47 % of which were encoded by one IME only. No antibiotic-resistance genes, bacteriocins, non-ribosomally synthesized or post-translationally modified peptides were detected on any of the ICEs and IMEs (ResFinder v3.1, Bagel4 [73]). ICE*Ms* ^NIBRBAC000500504^-2 carried putative resistance-genes for arsenic, cadmium, and mercury, while ICE*Ms* ^NIBRBAC000500504^-3 carried putative resistance-genes for mercury and chromate.

While the above analyses suggested that all ICEs and plasmids carry a highly variable spectrum of genetic cargo, a neighbor-joining tree based solely on the presence/absence of ICE accessory genes (Fig. S7) clearly separated ICESyms from other ICEs and plasmids, and also separated ICESyms based on their host-strain legume association. Comparative analysis of ICESym gene cargo with that of other ICEs identified genes specific to ICESyms (Table S5). As expected, these included genes coding for the synthesis and maturation of active nitrogenase (*nif* genes), for a bacteroid electron transfer flavoprotein complex and for a low O_2_ *cbb_3_-*type cytochrome oxidase (*fix* genes), genes participating in the biosynthesis and secretion of Nod factors (*nodABC* and *nodIJ*) and the recognition of plant flavonoids (*nodD2*). *fixV*, the key upstream regulator of the *nif* genes in R7A [67], was also present on all ICESyms. Conserved genes uninvestigated for roles in *Lotus*, *Cicer* and *Biserrula* symbiosis were also identified. A putative inosose isomerase with similarity to *Sinorhizobium meliloti* pSymB protein SMb20711 [82] was present in all ICESyms encoded adjacent to *nodD2*. Genes required for production of the queuosine precursor PreQ_0_, *queCDE*, were present on all ICESyms but not on other ICEs. Queuosine is a modified guanosine nucleotide base present on some tRNAs. In *S. meliloti *, *queC* appears to be required for an effective symbiosis with *Medicago trunculata* [83]. All *Lotus*-associated ICESyms also carry a second PreQ_0_ synthesis cluster with genes ordered *queEDC*. All ICESyms carried at least one copy of *dctA,* coding for a C_4_-dicarboxylate transport protein essential for nitrogen fixation [84]. In the *Lotus* lineage ICEs, a single copy of *dctA* was paired with cognate regulatory genes *dctBD*, while in the other ICESyms only *dctA* was present. The *Cicer*-associated ICEs and ICE*M*spSym^AA22^ harboured two copies of *dctA*. In *S. meliloti* pSymB, the expression of *dctA* is dependent on *dctBD* in free-living conditions but during symbiosis, *dctA* is efficiently expressed even in the absence of *dctBD* [85].

An association analysis was carried out comparing ICESym genes based on the host of isolation to identify genes that might be of specific adaptive significance for each host (Table S6). ICESym genes specifically associated with *Cicer*-associated mesorhizobia included the type III secretion system (T3SS) effector gene *nopP* and two hypothetical genes. These two hypothetical protein-coding genes are likely to code for T3SS effectors as they are preceded by T3SS boxes [86] and they were predicted to be secreted (SecP score > 0.73) by SecretomeP [87]. In *Bradyrhizobium* spp., variations in *nopP* alleles control soybean host compatibility [88]. All *Cicer*-associated ICESyms carry the nodulation gene *nodH* and a type II secretion system (T2SS) gene cluster. Using nhmmscan [66] and a hidden Markov model profile generated from characterised *nod* box sequences [28], we identified a putative *nod*-box motif upstream of the T2SS gene cluster in CC1192 (CP0150621 coordinates: 4,306, 615–4,306,661). Although the potential role of T2SS in symbiosis is not known, numerous phytopathogens employ T2SS systems for secretion of plant-tissue degrading enzymes [89]. It is possible that ICESym T2SS systems have a role in root colonization or infection. The ICESyms of *Lotus*-isolated mesorhizobia uniquely carry nodulation genes *nodS* (*msi131*), *noeJ* (*msi383*) and *noeK* (*msi384*), and an uncharacterised operon (*msi322* to *msi327*, and previously not-annotated EB234_30895 between *msi323* and *msi324*) encoding putative asparate and asparagine metabolism-related enzymes. No ICESym genes were significantly associated with strains specifically isolated from *Biserrula* hosts (Bonferroni *P*<0.05).

### Gene flux between MGEs

**Fig. 3.**
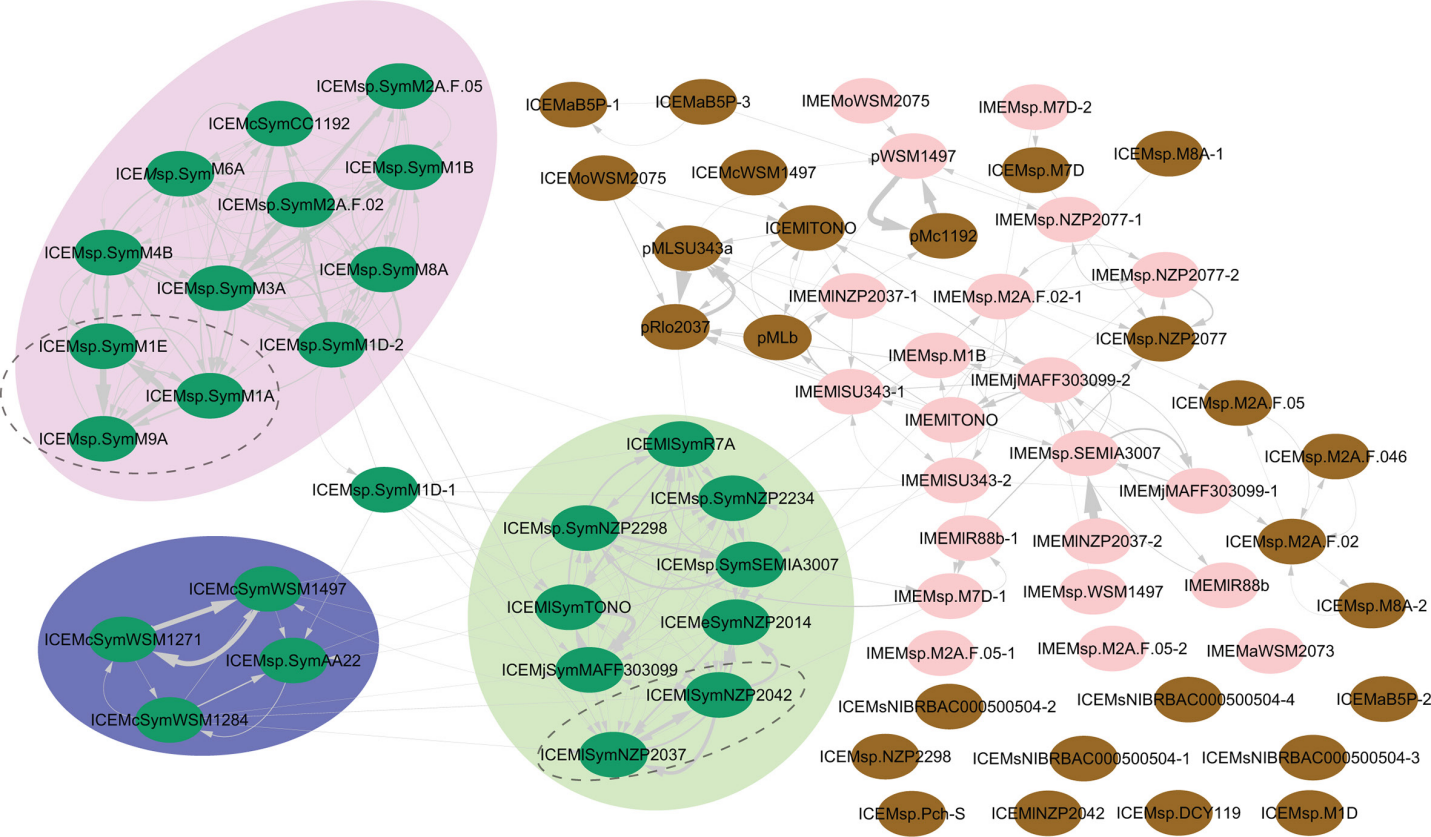
Recombination detected between and among ICEs, IMEs and plasmids. Cytoscape network produced with recombination analysis between ICE, IME and plasmid genes. Elements associated with brown circles are ICEs and plasmids lacking symbiosis genes, ICESyms are green while IMEs and the mobilizable plasmid pWSM1497 are pink. The coloured regions indicate highlight ICESyms associated with *Biserrula* (blue), *Lotus* (green) and *Cicer* (lilac).The tripartite ICEs are circled with a dashed line. Arrows between elements start from the query sequence used in Alfy nine and point to the element within which homologous regions were detected. Arrow width is proportional to the additive length of the sequences exhibiting homology.

ICEs can exchange genes with other MGEs through several mechanisms, including IS-mediated transposition and inter-ICE/IME/plasmid recombination [23, 90–92]. To detect evidence for inter-ICE recombination we used the neighbor-net algorithm [93] to create a network representing the relationship between the backbone genes present on ICEs and plasmids (Fig. S8). Patterns of reticulation within the network supported inter-ICE recombination even between highly conserved and contiguous ICE backbone genes (Phi test for recombination [94] was *P*<0.0001). Analyses with ClonalFrameML [65] also indicated recombination events between backbone genes. The alignment-free programme Alfy was used to extend the analysis to include entire gene content of ICEs, IMEs, and plasmids sequences [68]. Extensive evidence of gene exchange between ICEs and IMEs was detected. Clustering of ICEs based on evidence for gene exchange separated ICESyms from other ICEs and IMEs, suggesting greater gene exchange between ICESyms and little recombination between ICESyms and other ICEs or IMEs (Fig. 3). When this analysis was repeated using only contiguous gene clusters present on all ICESyms (Fig. S9), most of the connections indicative of DNA exchange between ICESyms reduced from 172 to 79, suggesting a large proportion of the detected recombination events represent homologous recombination between conserved ICESym regions.

**Fig. 4.**
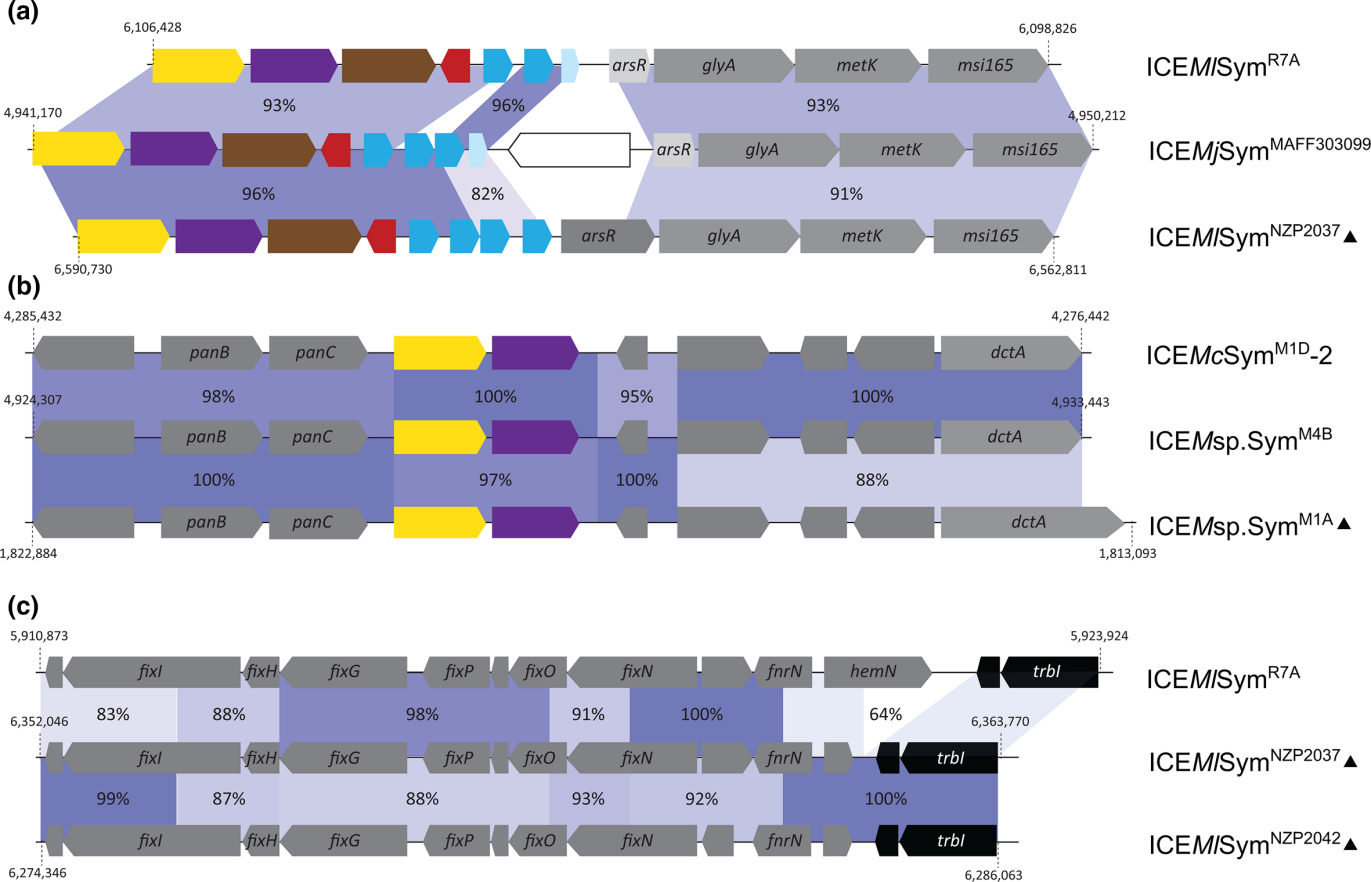
Chimaeric ICESym loci. Genes coding for QseC proteins are coloured in azure, *qseM* is displayed in red, *fseA* in brown, *traI* in purple, *traR* in yellow, and insertion sequences in white. Black indicates backbone genes, other genes are shown in grey, light-grey boxes indicate pseudogenes. Blue-shaded areas between genes on different ICESyms indicate homologous regions and the pairwise nucleotide identity is indicted. The triangle indicates tripartite ICE. (**a**) Comparison of the quorum-sensing-gene loci on ICE*Mj*Sym^MAFF303099^, ICE*Ml*Sym^NZP2037^ and ICE*Ml*Sym^R7A^. (**b**) Comparison of the quorum-sensing-gene loci on ICE*Mc*Sym^M1D^-2, ICE*M*sp.Sym^M4B^ and ICE*M*sp.Sym^M1A^. (**c**) Comparison of the *fix*-gene loci on ICE*Ml*Sym^NZP2037^, ICE*Ml*Sym^NZP2042^ and ICE*Ml*Sym^R7A^.

Given the extensive inter-ICESym recombination detected in the previous analyses, we interrogated some of the sequences in more detail. A comparison of the QS loci of the *Lotus* associated ICESyms ICE*Mj*Sym^MAFF303099^, ICE*Ml*Sym^NZP2037^ and ICE*Ml*Sym^R7A^, revealed several disruptions in average nucleotide identity between the three regions (Fig. 4a). In ICE*Mj*Sym^MAFF303099^, the nucleotide sequence between *traR2* and the second copy of *qseC* was 96 % identical to tripartite ICE*Ml*Sym^NZP2037^, however, downstream of the copy of *qseC* the nucleotide identity reduced to 82 % but shared 96 % identity to a region on ICE*Ml*Sym^R7A^. The QS locus in ICE*M*sp.Sym^M4B^ appeared to be a chimaera of the ICE*Mc*Sym^M1D^-2 and tripartite ICE*M*sp.Sym^M1A^, carrying alternating regions of 100 % pairwise nucleotide identity shared with these two ICEs (Fig. 4b). We also identified clear evidence for ancestral recombination between the *fix*-gene loci of ICE*Ml*Sym^NZP2037^, ICE*Ml*Sym^NZP2042^ and ICE*Ml*Sym^R7A^ (Fig. 4c).

### The largest documented horizontally mobile ICE, ICE*Msp*Sym^AA22^

*Mesorhizobium* sp. AA22 was isolated from *Biserrula pelecinus* and carries a tripartite ICE with a total length of 853, 775 bp, making ICE*M*spSym^AA22^ the largest ICE identified in any bacterium to date. The α, β and γ fragments are 766, 834, 70,134 and 16, 815 bp in length respectively. The other tripartite ICEs have α, β and γ fragments sized between 443,551 and 563, 069 bp, between 19, 361 and 74, 018 bp, and between 5,412 bp and 25,603 bp respectively, indicating that most of the expansion in ICE*M*spSym^AA22^ size has occurred within the α fragment. To determine if ICE*M*spSym^AA22^ was mobile, we carried out conjugation experiments using the ICE-cured *M. japonicum* R7A derivative R7ANS as a recipient [32]. ICE*M*spSym^AA22^ transferred with an average frequency of 6.0×10^−8^ exconjugants per donor (standard deviation 2.3×10^−8^). An exconjugant was isolated and sequenced using short-read sequencing. *De novo* assembly (NZ_NSFP00000000) confirmed each of the *attL* and *attR* site junctions were present and mapping of the exconjugant reads to the AA22 sequence confirmed the entire ICE*M*spSym^AA22^ had transferred and recombined with the R7ANS chromosome as expected [26].

Pangenome analysis highlighted several gene clusters unique to ICE*M*spSym^AA22^. For instance, ICE*M*spSym^AA22^ harbours a complete *nuo* (NADH:ubiquinone oxidoreductase) gene cluster located at the beginning of the α fragment. The ICE*M*spSym^AA22^ *nuo* cluster is distinct from that coded for on the AA22 chromosome both in gene order and nucleotide identity (maximum identity observed of 59.6 % for *nuoM*). Interestingly, the ICE*Msp*Sym^AA22^ *nuo* cluster shares the same gene arrangement and 81.7 % average pairwise nucleotide identity to that encoded on the symbiosis plasmid pSymA of *S. meliloti* [95].

ICE*M*spSym^AA22^ is also the only ICE in *Mesorhizobium* to carry a CRISPR-Cas system. The type I-C CRISPR-Cas system (CP048406.1, coordinates: 6, 182,785–6,192, 544) harbours *cas3*, *cas5*, *cas8* and *cas7*; however it lacks *cas4*, *cas1* and *cas2* involved in spacer acquisition [96, 97]. The CRISPR locus harbours over 40 spacers interspersed with non-identical palindromic repeats. The targets of the spacers (identified via blastn and CRISPRTarget [98]) appear similar to putative phage and plasmid sequences, some of which are found in other soil bacteria such as *Mesorhizobium, Rhizobium, Streptomyces, Azospirillum, Agrobacterium* and *Paraburkholderia *.

## Conclusions

In this work we analysed 41 complete *Mesorhizobium* spp. genomes to enumerate the conjugative and mobilizable elements present. Of the pangenome genes present on an identifiable MGE (12 %), more than 70 % were present across 56 ICEs related to ICE*Ml*Sym^R7A^, including the largest mobile ICE characterised to date, ICE*M*sp.Sym^AA22^. The remaining mobilome spanned 24 plasmids, 27 IMEs and 17 bacteriophages. On average, each genome carried at least one ICE (1.22±0.85) and 50 % of strains carried an IME. In contrast, of the 41 genomes examined, only 16 carried plasmids and only seven genomes harboured multiple plasmids.

The ICESyms formed a clear monophyletic group distinct from the other identified mesorhizobial ICEs. Presumably, following the capture of symbiosis genes by an archetypal ICESym, descendants diverged into ICESyms that specify symbiosis with different legumes. It is unclear if this archetypal ICESym evolved within the *Mesorhizobium* genus or was acquired from outside the genus. It is also unclear why symbiosis plasmids, which are the most common form of symbiosis MGE in other rhizobia, are rarely identified in *Mesorhizobium* spp. In *Rhizobium* spp. symbiosis plasmids predominantly carry *vir*-like conjugation systems [99] and interestingly, the only conjugative mesorhizobial symbiosis plasmid identified in this analysis (in CCNWGS0123) also carries a *vir*-like conjugation system. Thus there seems to be no intrinsic barrier to establishment of these conjugative symbiosis plasmids in *Mesorhizobium *. Broader genomic comparisons suggest ICEs generally have a larger host range than their plasmid counterparts [100], so it may be that once established in *Mesorhizobium* spp., ICESyms have been more successful infiltrating diverse members of the genus. Regardless of the mechanistic or evolutionary reasons, it is clear from these analyses that ICEs dominate the mobilome of nitrogen-fixing legume symbionts of the *Mesorhizobium* genus.

The ICEs and IMEs identified in this work were found integrated within 16 distinct sites in the chromosome and carried an overlapping set of integrase genes and integration sites. The phylogeny of ICE backbone genes suggests some ICEs have switched between integration sites during evolution and some ICEs even appear to have become plasmids in several branches of the tree (e.g. pMLb and pCC1192). Overall these observations mirror those made from broader analyses of ICEs, IME and plasmids in bacteria [100]. Given the ubiquity of conjugative elements identified in these genomes, we suspect that the frequent switching of integration mechanisms by these elements reflects fierce competition for chromosomal integration sites amongst ICEs and IME in this genus.

There is clear evidence that some ICESyms have likely switched from a monopartite to a tripartite structure. The tripartite-ICE integrase genes specifying integration at tRNA-Met(a) and *guaA* are distinct from their closest counterparts on monopartite ICEs (Fig. S5) suggesting the tripartite ICE recombination system evolved once. The observation that the tripartite ICEs structure appears a multiple positions in the ICE backbone tree suggests that tripartite ICEs have likely recombined with monopartite ICESyms and replaced the recombination systems on these ICEs.

We identified numerous instances of gene duplications, pseudogenization, rearrangements and recombination between loci involved in quorum sensing (*traR* and *traI*) and its inhibition (*qseM* and *qseC*) (Figs 2 and 4). In contrast, the gene organisation of conjugation-gene clusters and *rdfS*, *traF* and *rlxS* genes remained remarkably conserved even between very distantly related plasmids and ICEs. The frequent rearrangements of top-level regulators of transfer may reflect the conflicting evolutionary forces faced by ICEs, which must balance their evolutionary trajectories between vertical and horizontal descent. High frequency ICE transfer may increase the likelihood that an ICE will arrive in a new host with superior attributes but high transfer rates could also tax the bacterial host resources and hence be subject to negative selection. Dysregulation of quorum sensing resulting from two ICESyms in the same cell might also drive the observed rearrangements. Consistent with this possibility, introduction of an extra copy of *traR* and the preceding promoter region on a low-copy plasmid is enough to activate AHL production, ICE*Ml*Sym^R7A^ excision and conjugative transfer in *M. japonicum* R7A [32, 33]. Interestingly, in the only strains in this dataset carrying two ICESyms (* Mesorhizobium* sp. M1D.F.Ca.ET.043.01.1.1 and M2A.F.Ca.ET.046.03.2.1) one of these ICESyms, ICEMsp.Sym^M1D^-1, is also the only ICESym lacking a copy of the *traI1* AHL-synthase gene (Fig. 2b).

Our analysis of gene flux between MGE suggests a substantial amount of homologous recombination occurs between the ICESyms and in particular, ICESyms specifying symbiosis with the same legume host. There are several explanations why recombination may occur more readily between the ICESyms than other ICEs. More closely related ICESyms are more likely to utilise the same integration sites and therefore integrate in tandem in the same locus, a situation known to promote ICE recombination [90]. The frequency of homologous recombination is exponentially more likely to occur between the closely-related sequences of the ICESyms (88 % backbone pairwise nucleotide identity) than with less related ICEs (69 % backbone pairwise nucleotide identity) [101]. Mesorhizobia that participate in symbiosis with the same legume host may occupy the same niche, providing greater opportunity for ICESyms to encounter each other in the same cell through HGT. An interesting observation from the comparisons of conserved ICE backbone genes is that all ICESym and ICE*M*sp.^M2A.F.046^ lack the *trbK*, tentatively indicating ICESyms have lost one mechanism to inhibit entry of competing ICESyms. This could suggest that homologous recombination has been an integral part of ICESym evolution and that loss of *trbK* has contributed by increasing the opportunity for two ICESyms to meet in the same cell.

## Supplementary Data

Supplementary material 1

Supplementary material 2
