# Supplementary material for: Comparative analysis of integrative and conjugative mobile genetic elements in the genus Mesorhizobium
Source: Microb Genom. 2021 Oct 4;7(10):000657. doi: 10.1099/mgen.0.000657 (PMC8627217; doi:10.1099/mgen.0.000657)
Supplement: Supplementary material 1 [file mgen-7-0657-s001.pdf]

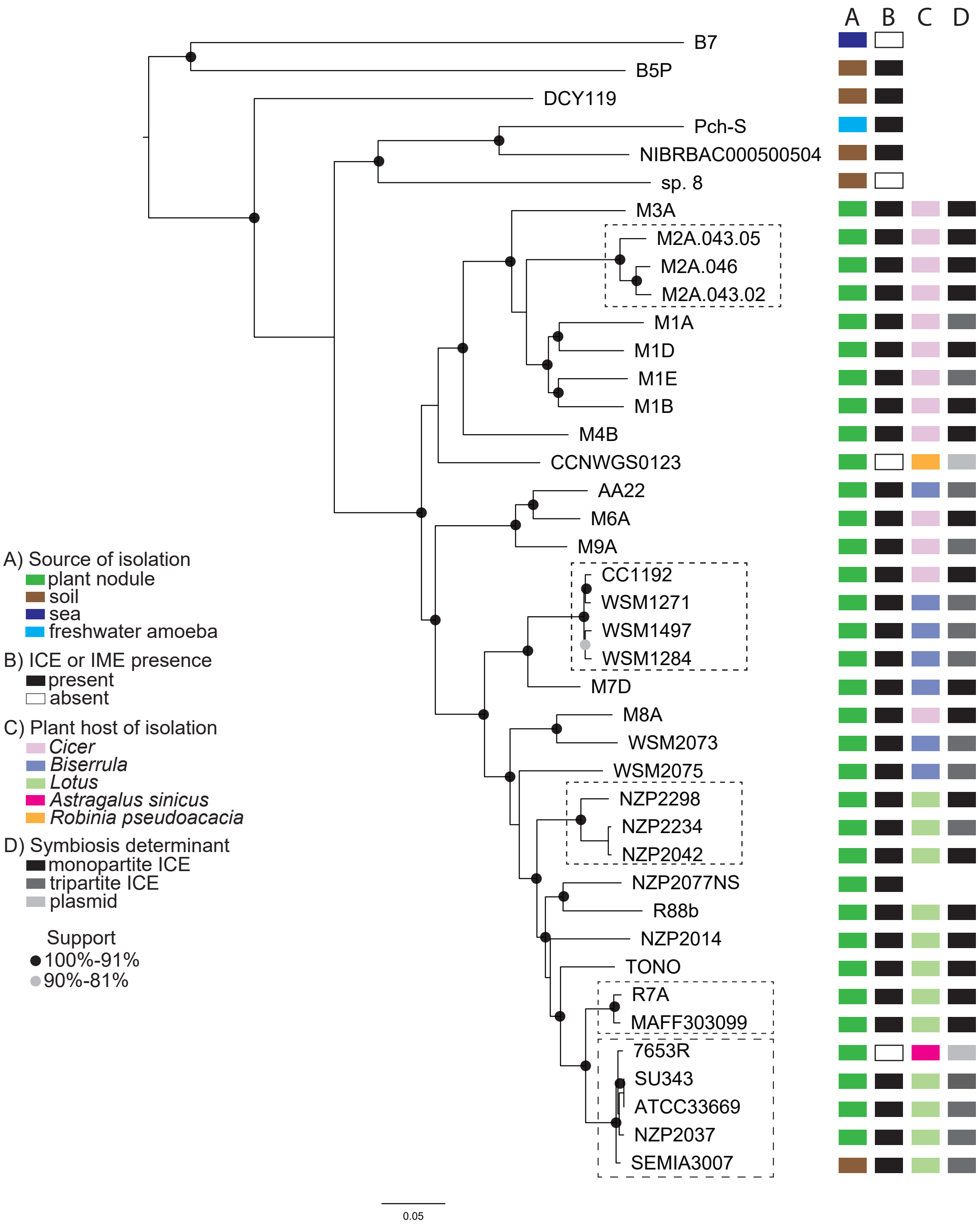

Figure S1. Core genome phylogeny of the *Mesorhizobium* strains analysed in this study. A maximum likelihood phylogeny of the 41 strains used in this study was constructed from a concatenated alignment of 1,609 single-copy core genes. The tree was rooted at midpointwith. The scale represents the mean number of nucleotide substitutions per site. Strains showing an ANI ≥ 95% are grouped in dashed rectangles.

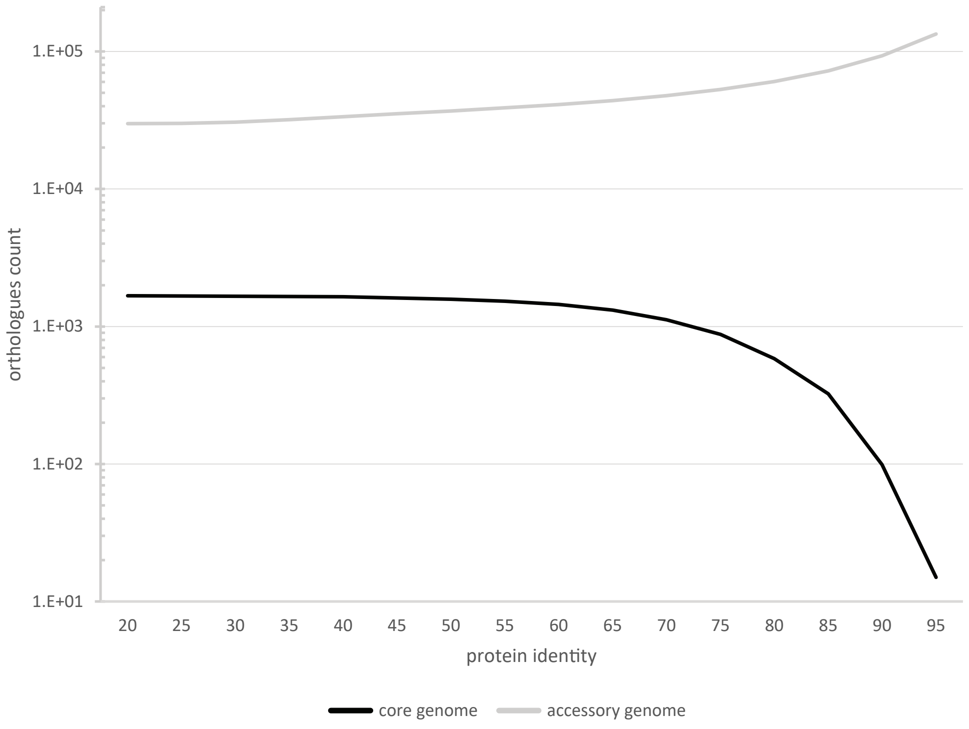

Figure S2. Pangenome calculations. Number of orthologues in the accessory and core genome of the 41 *Mesorhizobium* genomes calculated incrementing the protein identity threshold by which proteins are classified as same orthologue.

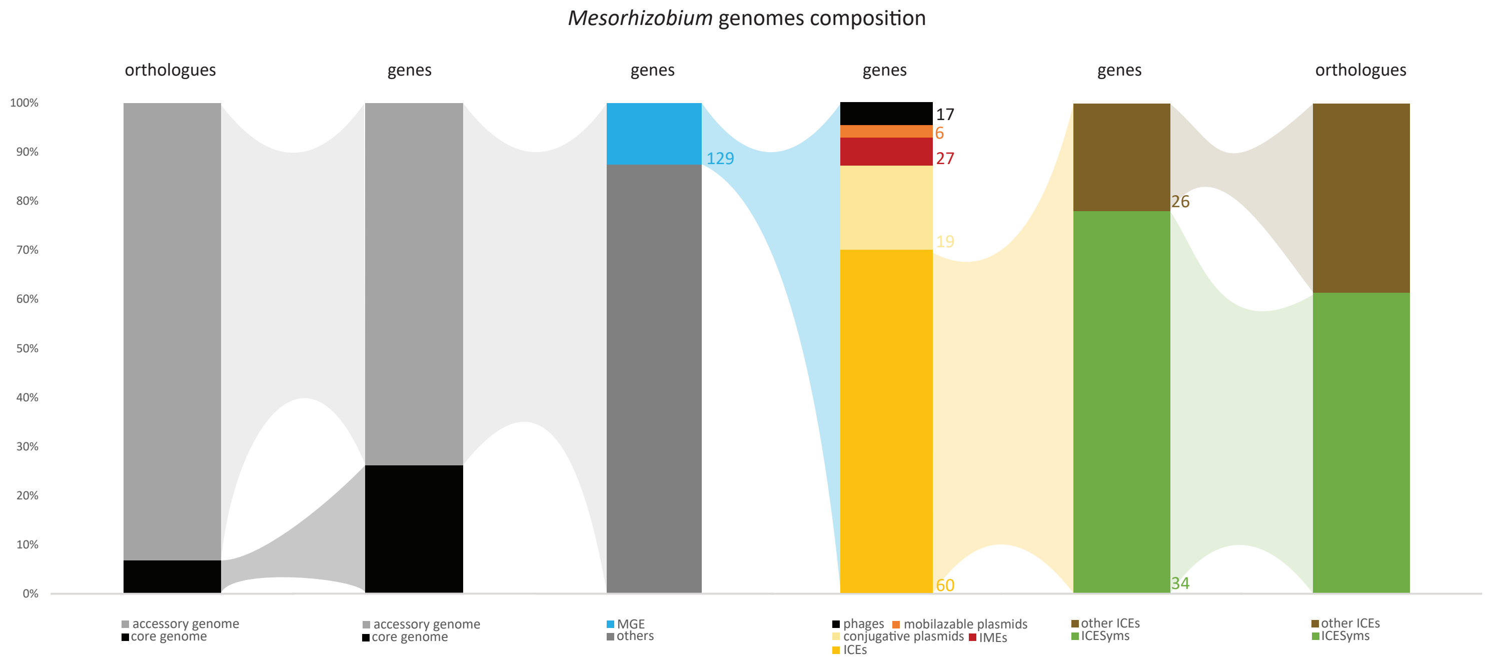

Figure S3. *Mesorhizobium* genome composition. Proteinortho was used to classify the number of orthologues and genes present in the core and accessory genome of the 41 *Mesorhizobium* genomes. The genomes shared a core and an accessory genome of 1,671 and 22,947 orthologues, respectively, which corresponds to a total of 68,731 genes of core genome and 193,100 genes of accessory genome. Genes that were present on recognised mobile elements (Table S2) were classified as MGE, which were divided in ICEs, IMEs, conjugative and mobilizable plasmids, and phages. Finally, the percentage of genes and orthologues present in ICESyms and the other ICEs are reported. Numbers on the right of the bar refer to the number of elements that fall in that category.

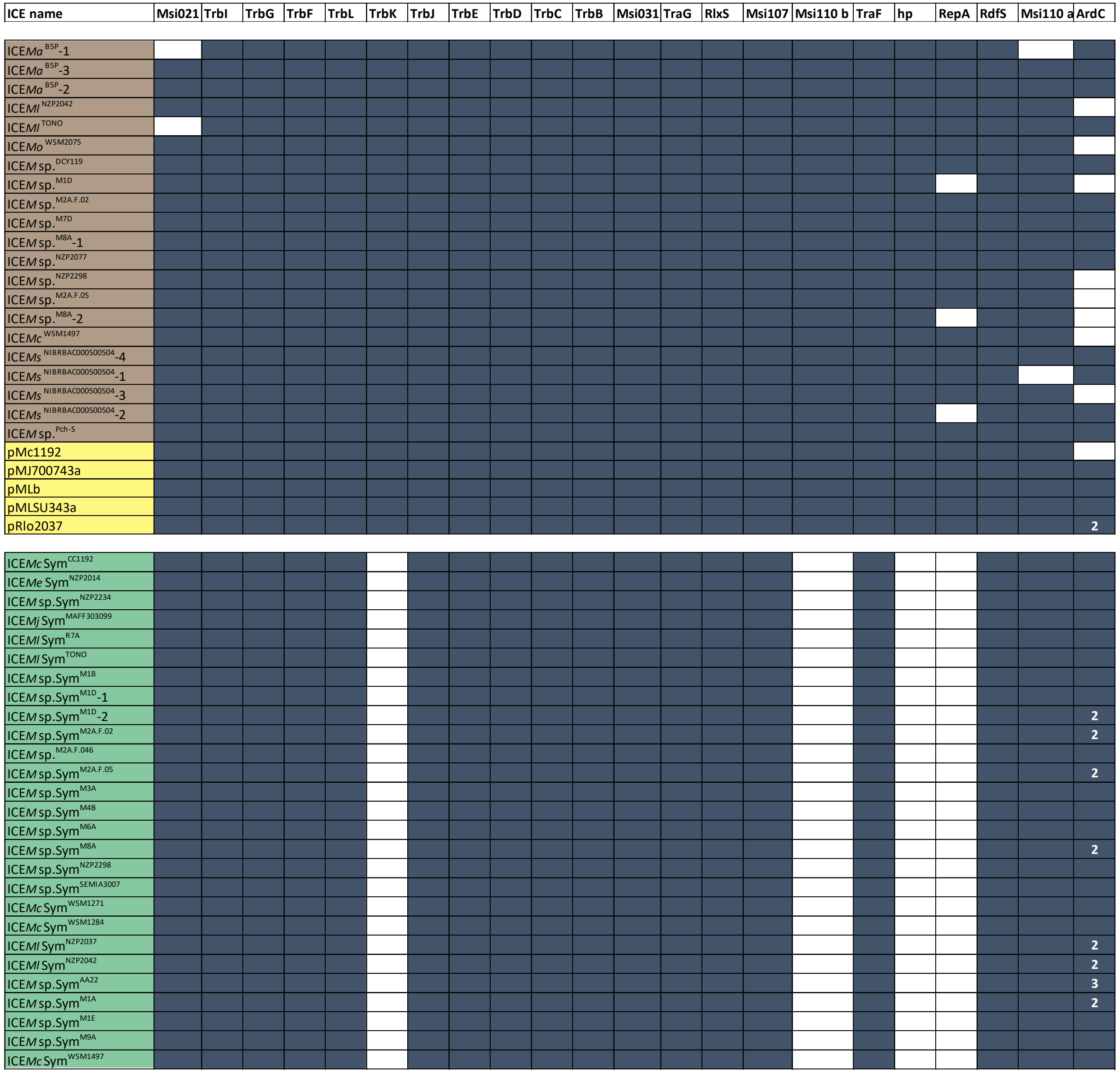

Figure S4. Presence and absence of backbone genes in the ICEs and plasmids analysed in this study. The name of the elements are highlighted in brown and yellow for ICEs and plasmids lacking symbiotic genes, respectively, and in green for ICESyms and ICEMsp.M2A.F.046. Boxes are in navy blue if the gene is present, in white if the gene is absent, the number inside the boxes indicates number of copies if more than one.

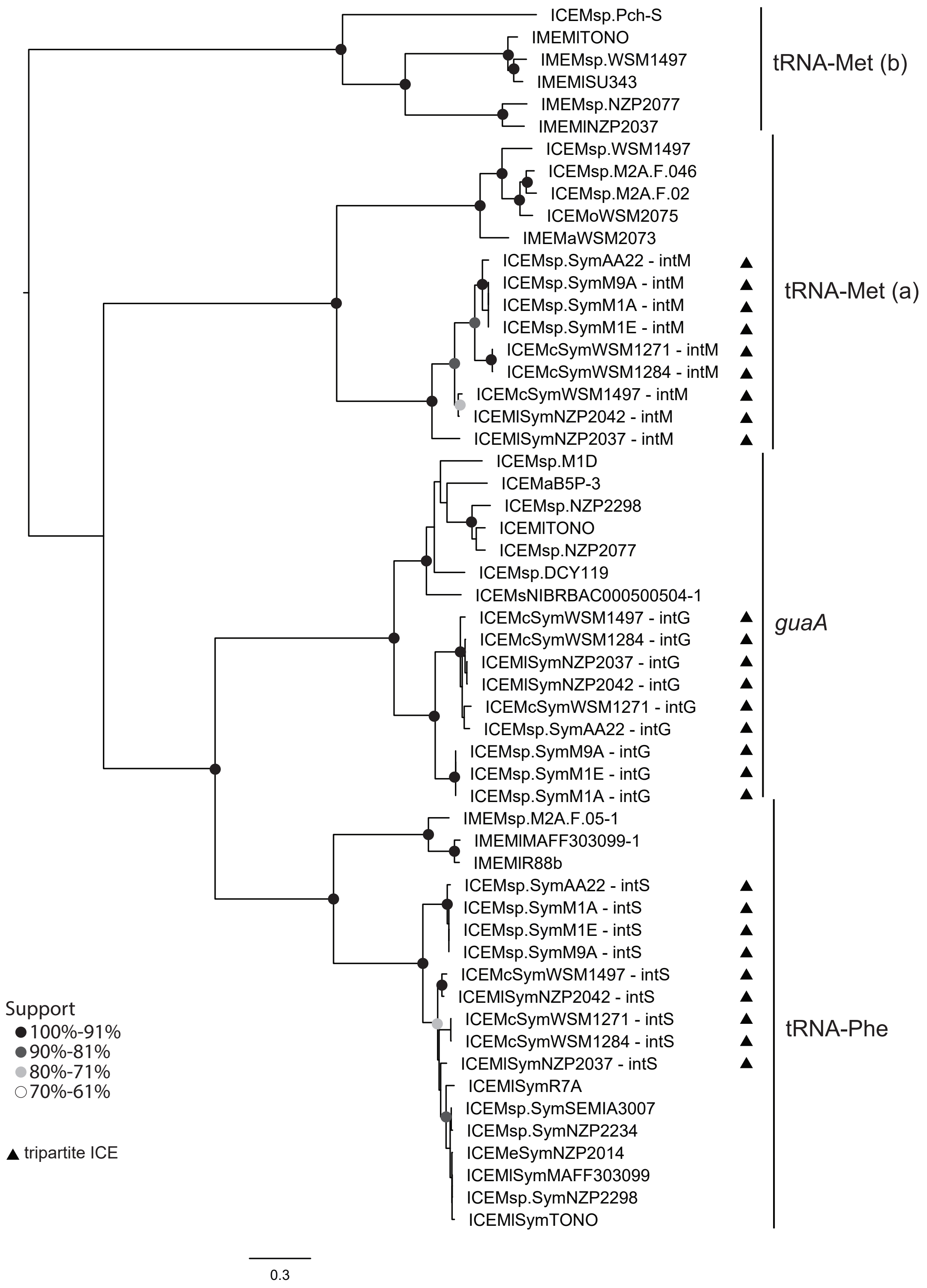

Figure S5. RAxML tree of integrases specifying integration in guaA, tRNA-Met and tRNA-Phe. For each clade of integrases, the integration site is reported on the side.

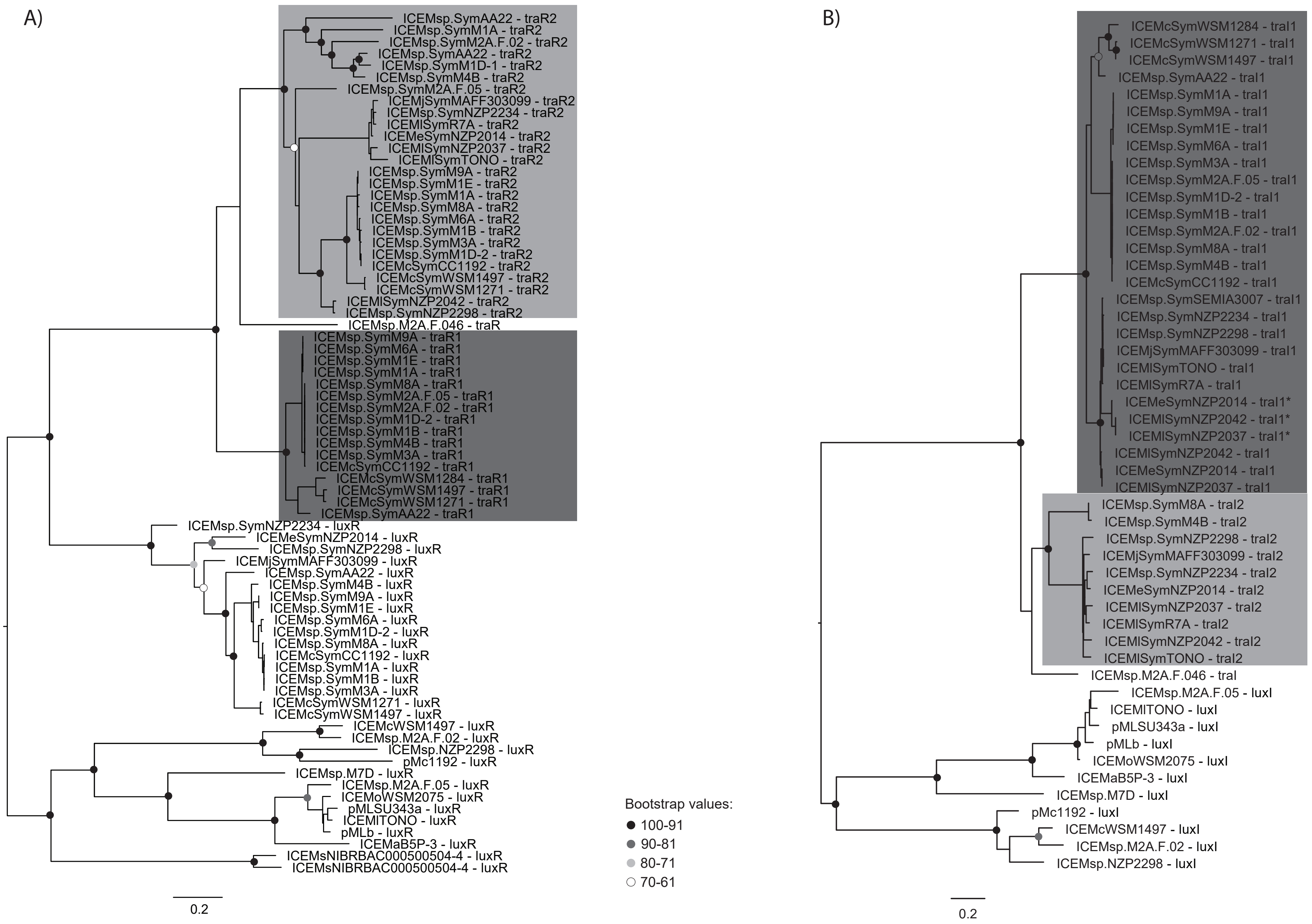

Figure S6. RAxML tree of the quorum-sensing genes. A) Tree of *traR* and *luxR*, and B) tree of the *traI* and *luxI* genes present in the ICESyms, ICEs and plasmids were rooted at midpoint. Groupings represent homologues genes in the ICESyms.

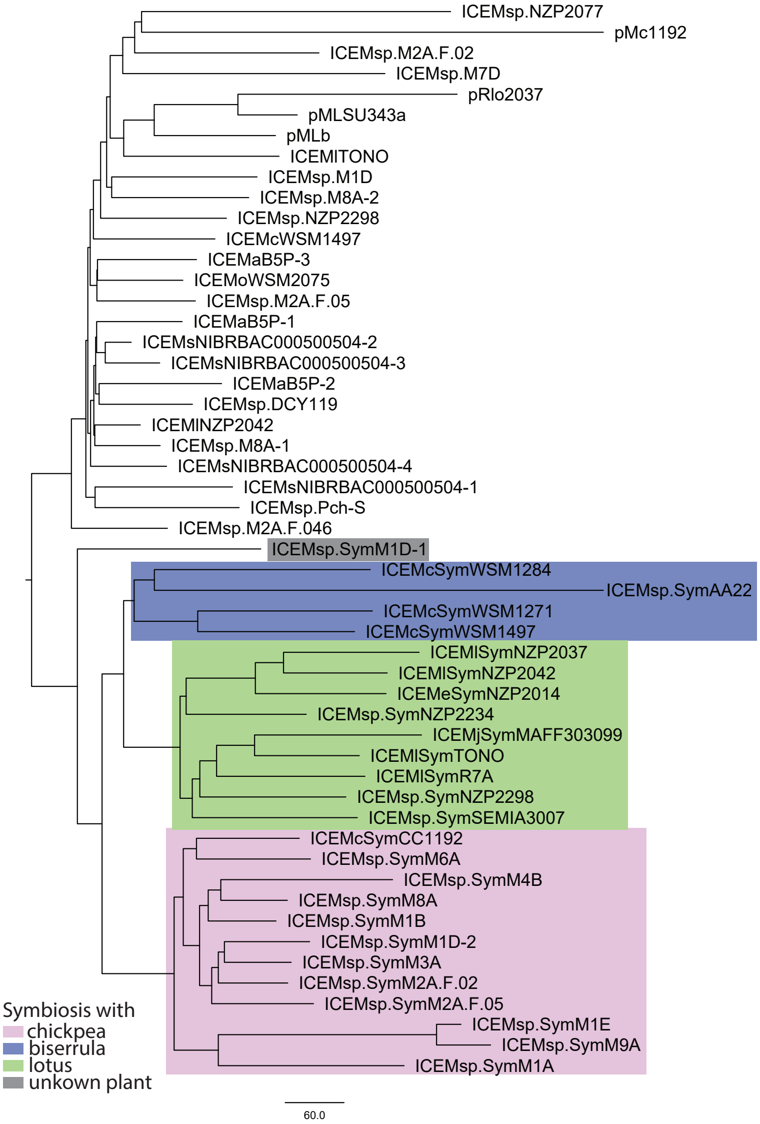

Figure S7. Accessory genes of ICEs. Neighbor-joining tree based on the presence of accessory genes in *Mesorhizobium* ICEs produced by Roary run using a minimum amino-acid identity of 40%. The tree was rooted at midpoint.

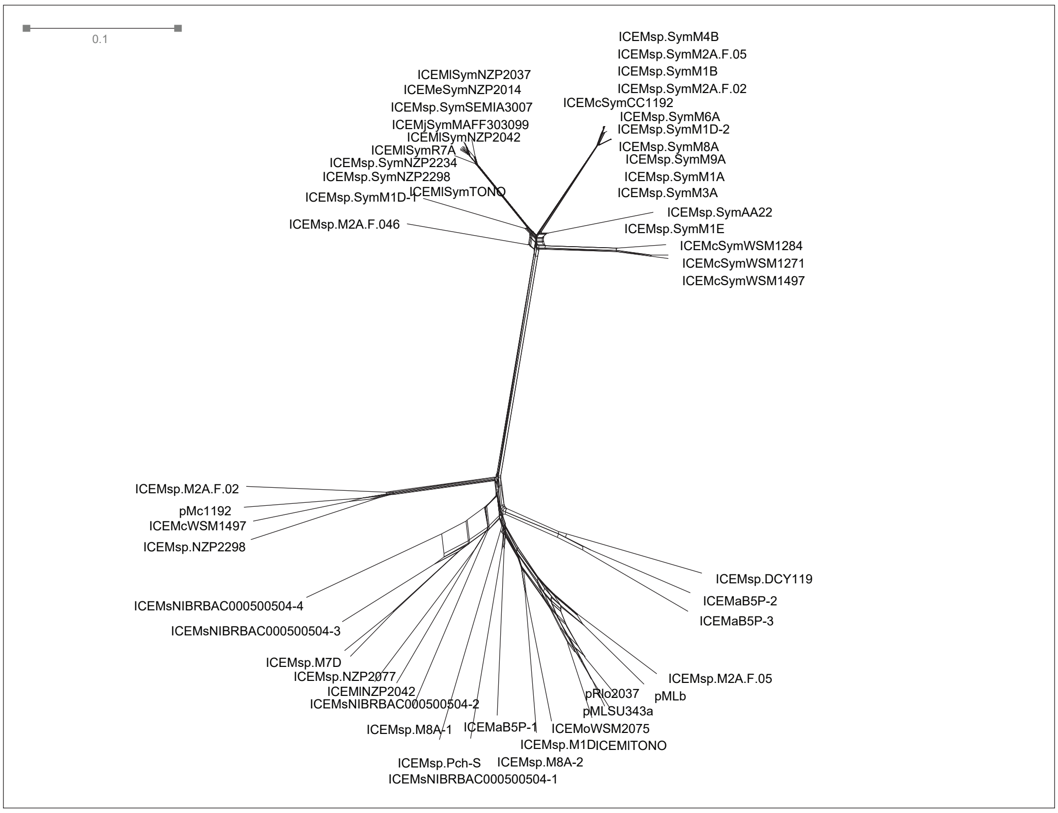

Figure S8. Neighbor-net network tree of backbone genes of ICEs. The tree produced with SplitTree is based on the concatenation of the alignment of conserved backbone genes of the elements used for Figure 2A.

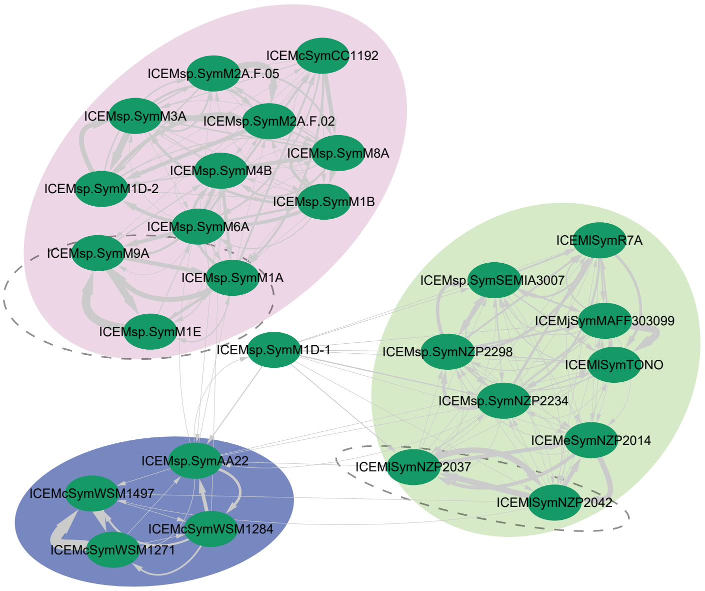

Figure S9. Recombination detected between among "minimal" ICESyms. Cytoscape network produced with recombination analysis between "minimal" ICESyms genes. The coloured regions indicate highlight ICESyms associated with *Biserrula* (blue), *Lotus* (green) and *Cicer* (lilac).The tripartite ICEs are circled with a dashed line. Arrows between elements start from the query sequence used in Alfy 9 and point to the element within which homologous regions were detected. Arrow width is proportional to the additive length of the sequences exhibiting homology.
